# A Comparative Biochemical and Pathological Evaluation of Brain Samples from Knock-In Murine Models of Gaucher Disease

**DOI:** 10.3390/ijms25031827

**Published:** 2024-02-02

**Authors:** Makaila L. Furderer, Bahafta Berhe, Tiffany C. Chen, Stephen Wincovitch, Xuntian Jiang, Nahid Tayebi, Ellen Sidransky, Tae-Un Han

**Affiliations:** 1 Medical Genetics Branch, National Human Genome Research Institute, National Institutes of Health, Bethesda, MD 20892, USA; kailafurderer@gmail.com (M.L.F.); bahafta.berhe@nih.gov (B.B.); tiffany.chen2@nih.gov (T.C.C.); tayebin@mail.nih.gov (N.T.); 2 Aligning Science Across Parkinson’s (ASAP) Collaborative Research Network, Chevy Chase, MD 20815, USA; 3 Advanced Imaging & Analysis Core, National Human Genome Research Institute, National Institutes of Health, Bethesda, MD 20892, USA; wincovis@mail.nih.gov; 4 Washington University Metabolomics Facility, Washington University School of Medicine, St. Louis, MO 63110, USA; jiangxuntian@wustl.edu

**Keywords:** Gaucher disease, glucocerebrosidase, neuropathology, glucosylsphingosine, murine models, Parkinson’s disease

## Abstract

Gaucher disease (GD) is a lysosomal storage disorder stemming from biallelic mutations in *GBA1*, characterized by glucocerebrosidase dysfunction and glucocerebroside and glucosylsphingosine accumulation. Since phenotypes of murine models of GD often differ from those in patients, the careful characterization of *Gba1* mutant mice is necessary to establish their ability to model GD. We performed side-by-side comparative biochemical and pathologic analyses of four murine *Gba1* models with genotypes L444P/L444P (p.L483P/p.L483P), L444P/null, D409H/D409H (p.D448H/p.D448H) and D409H/null, along with matched wildtype mice, all with the same genetic background and cage conditions. All mutant mice exhibited significantly lower glucocerebrosidase activity (*p* < 0.0001) and higher glucosylsphingosine levels than wildtype, with the lowest glucocerebrosidase and the highest glucosylsphingosine levels in mice carrying a null allele. Although glucocerebrosidase activity in L444P and D409H mice was similar, D409H mice showed more lipid accumulation. No Gaucher or storage-like cells were detected in any of the *Gba1* mutant mice. Quantification of neuroinflammation, dopaminergic neuronal loss, alpha-synuclein levels and motor behavior revealed no significant findings, even in aged animals. Thus, while the models may have utility for testing the effect of different therapies on enzymatic activity, they did not recapitulate the pathological phenotype of patients with GD, and better models are needed.

## 1. Introduction

Gaucher disease (GD), a prevalent lysosomal storage disorder, is caused by biallelic mutations in *GBA1*, the gene encoding the lysosomal enzyme glucocerebrosidase (GCase). GCase is responsible for catalyzing the hydrolysis of the glycolipids glucosylceramide (GlcCer) and glucosylsphingosine (GlcSph). When GCase activity is deficient, these lipid substrates accumulate, resulting in engorged macrophages known as Gaucher cells, a hallmark of the disease. Mutations in *GBA1* are also the most common risk factor for the development of Parkinson’s disease (PD). Clinical studies noting patients with GD and early onset PD led to the discovery of this intriguing relationship [1,2]. There is a broad range of clinical presentations in GD, extending from severe and rapidly progressive neurodegeneration in infancy to patients with extensive skeletal or visceral disease to largely asymptomatic cases identified in the elderly [3,4]. Animal models that accurately reproduce the phenotypes observed in the different types of human GD are essential for understanding this heterogeneity, as well as for developing and testing the efficacy of novel therapeutics [5].

Mouse models of GD have been challenging to generate as they often fail to produce the same phenotype observed in human disease [6,7]. A total knock-out of glucocerebrosidase leads to a neonatal lethal phenotype with impaired skin ultrastructure, analogous to the most severe neonates with type 2 GD [8]. Fortunately, other strategies have been used to establish models that more closely resemble most patients with acute neuronopathic GD (nGD) or chronic nGD. The K14-lnl/lnl line was developed to circumvent the lethal skin phenotype observed in other KO models [9]. Neuropathological evaluation confirmed prominent abnormalities, making this a useful model for testing therapeutics. Pewzner-Jung et al. (2021) developed a mouse model by utilizing doxycycline to regulate the expression of a *Gba1* transgene to generate *Gba*^−/−^: *Gbatg* mice that phenotypically modeled type 3 GD, displaying GlcCer accumulation, neuronal loss and gliosis [10]. Several other nGD models were also generated, but the mice either died perinatally or lack of symptoms analogous to those seen in patients with GD [11]. In addition to the failure of many of the models to recapitulate human GD phenotypes, some available models such as D409V (D448V) mice do not carry mutations commonly found in patients [11].

In humans, homozygosity for the L444P (p.L483P) mutation is usually associated with chronic nGD [3]. Neuronal loss and degeneration are the most common neuropathological features of human nGD [12,13]. Early attempts to create a L444P murine model by introducing a duplicated mutant allele resulted in mice that died after 1–2 days [14]; subsequently, it was shown that mice homozygous for L444P with the WT version of the *Ugcg* gene actually had a normal lifespan [15]. Several studies have established that while GCase activity in homozygous mice is ~20% of WT [14,15,16], the mice do not accumulate GlcCer. Associated symptoms include inflammation, anemia, leukopenia, skin abnormalities and reduced serum cholesterol levels [15]. Crossing these L444P mice with a *Gba1* KO model resulted in *Gba^L444P/null^* mice, which had greatly reduced GCase enzyme activity, and elevated GlcSph levels but showed no GlcCer accumulation [17]. A second L444P/L444P mouse was generated via targeted homologous recombination. While these mice have a normal lifespan and breed well, observed histopathology included Gaucher cells in viscera and mild astrogliosis [18]. Data on behavioral testing, especially regarding motor function, on any L444P homozygous mice have not been published.

The D409H (p.D448H) is another severe GD mutation. GCase with mutation D409H is catalytically defective, susceptible to proteolytic digestion, and unstable GCase [19]. In humans, homozygosity for D409H induces a unique phenotype consisting of oculomotor abnormalities, hydrocephalus and valvular/aortic calcifications [20,21]. Unfortunately, mice with genotypes D409H/D409H and D409H/null failed to display the phenotype detected in human GD patients. Mice homozygous for D409H lack central nervous system (CNS) pathology and have a normal lifespan [13,22]. Low levels of GlcCer accumulation in tissues other than the brain were observed [19,22]. Subsequently, Xu et al. (2003) crossed homozygous D409H/D409H animals with null/WT animals to produce D409H/null mice. Gaucher cells in the spleen lungs and liver appeared 3–4 months earlier in the D409H/null animals, but no GlcCer accumulation was observed in the brain in either model, and they did not display CNS abnormalities phenotypically or histologically [19].

An ongoing need in this field is an appropriate mouse model for assessing the impact of drug interventions targeting the brain. Since it is difficult to compare mutant mice that were generated with diverse mutations and strategies, and in different facilities, we decided to evaluate a panel of mice in parallel using the same breeding and caging conditions, animal care and pathology assessments, focusing on a direct comparison of the L444P and D409H homozygous models in our colony. In addition, through cross breeding, we generated compound heterozygotes with either a L444P or D409H allele and a null allele. We studied the phenotypes of mice with genotypes wildtype (WT), *Gba^L444P/L444P^* (LP/LP), *Gba^D409H/D409H^* (DH/DH), *Gba^L444P/null^* (LP/null) and *Gba^D409H/null^* (DH/null) side-by-side, evaluating markers of astrogliosis, microgliosis, the density of dopaminergic neurons and α-synuclein levels and aggregation. Some lines also underwent behavioral testing. Based on data from previous studies, we hypothesized that the haploinsufficient/point mutant mice might better model chronic neuronopathic GD- or *GBA1*-associated PD and have enhanced utility for therapeutic development.

## 2. Results

### 2.1. Evaluation of GCase Pathology in Gba1 Mutant Mice

**Figure 1:**
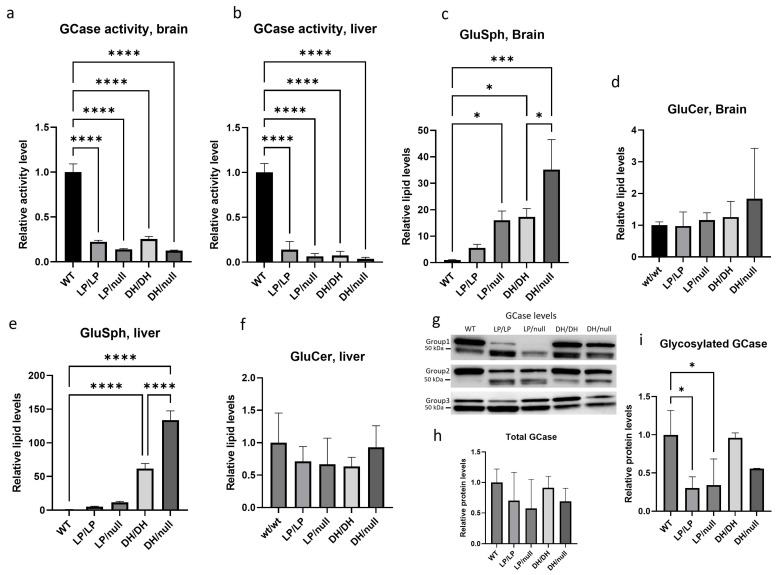
Evaluation of glucocerebrosidase activity, protein levels and glycolipid levels in *Gba1* mutant mice. All assays were conducted in tissue lysates from either wildtype (WT), *Gba^L444P/L444P^* (LP/LP), *Gba^L444P/null^* (LP/null), *Gba^D409H/D409H^* (DH/DH) and *Gba^D409H/null^* (DH/null) animals at age six months. (**a**) Relative GCase activity in brain lysates. (**b**) Relative GCase activity in liver lysates. (**c**) Relative levels of glucosylsphingosine in brain lysates. (**d**) Relative levels of glucosylceramide in brain lysates. (**e**) Relative levels of glucosylsphingosine in liver lysates. (**f**) Relative levels of glucosylceramide in liver lysates. (**g**) Immunoblotting for evaluation of GCase levels in RIPA using brain lysates. (**h**) Quantification of immunoblot band intensity for combined bands. (**i**) Quantification of immunoblot band intensity for the high-molecular-weight band. Intensity of each immunoblot band was normalized by total protein of the same lane measured with Ponceau-S staining. All the assays were repeated in three different animals for each genotype group and then averaged. Error bars indicate the standard deviation. One-way ANOVA with Sidak’s multiple-comparison test was the statistical method used. * *p* < 0.05; *** *p* < 0.001; **** *p* < 0.0001.

GCase activity was significantly reduced in LP/LP and DH/DH mice (Figure 1a,b) in both the brain and the liver. The combination of a null allele and a point mutation resulted in lower GCase activity than that seen in homozygous L444P and D409H point mutations in brain lysates, and this trend was also observed in liver lysate, although neither reached statistical significance. The fluorescence and enzyme activity assay data are available on Zenodo at https://zenodo.org/records/10431006 (accessed on 25 December 2023). Lipid analysis showed that none of the models accumulated GlcCer (Figure 1d,f). Each of the *Gba1* mutant models had higher levels of GlcSph than WT in both brain and liver (Figure 1c,e). The LP/LP and DH/DH mice showed 6-fold and 17-fold increases in GlcSph in the brain, respectively, while the LP/null and DH/null mice showed 16-fold and 35-fold increases in GlcSph in the brain, respectively. These data indicate that in these models, the D409H mutation has a greater impact on GlcSph accumulation than the L444P mutation, and that the combination of a null allele and point mutations increased the lipid accumulation compared to mice with homozygous point mutations. This trend was also observed in the liver, where the impact of D409H on GlcSph accumulation was greater than L444P, and hepatic GlcSph levels were higher than in the brain (Figure 1e). The lipid analysis data are available on Zenodo at https://zenodo.org/records/10431187 (accessed on 25 December 2023).

Immunoblotting showed double bands for the GCase protein, where the upper band was around 60 kDa, which corresponds to the molecular weight of glycosylated GCase, and the lower 50 kDa band likely represents deglycosylated GCase. The combined quantification of both bands showed no significant difference in total GCase levels between the different *Gba1* genotypes (Figure 1h). However, quantification of the glycosylated GCase band showed that the GCase levels in LP/null and LP/LP were significantly lowered than wildtype GCase (Figure 1i), while in DH/DH and DH/null mice, the levels were not different from wildtype (Figure 1i). These data indicate that the GCase protein levels alone are not driving the GlcSph levels. Also, the Western blots suggest that the L444P mutant form of GCase is more deglycosylated than wildtype GCase. All the immunoblot quantification data are available on Zenodo at https://zenodo.org/records/10431048 (accessed on 25 December 2023).

### 2.2. Evaluation of Visceral Pathology of Gba1 Mutant Mice

**Figure 2:**
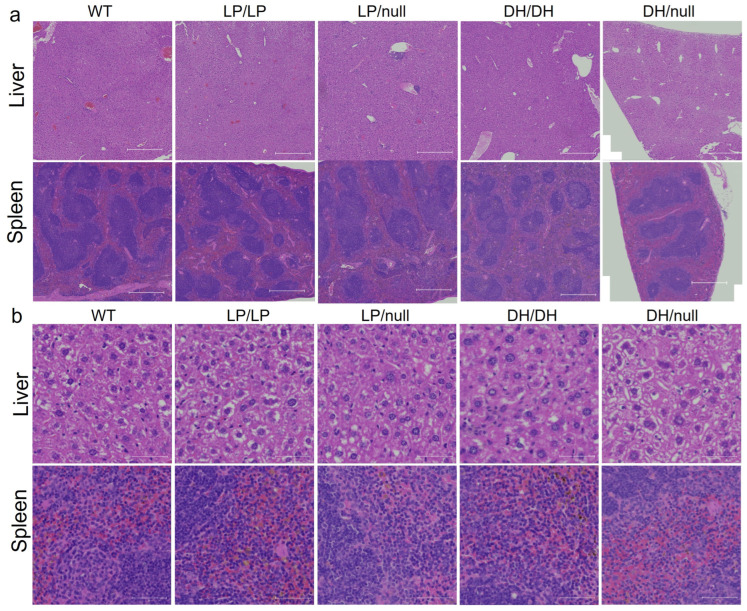
Histological evaluation of *Gba1* mutant mice in liver and spleen. H&E staining was conducted on transverse section of liver or spleen tissue from wildtype (WT), *Gba^L444P/L444P^* (LP/LP), *Gba^L444P/null^* (LP/null), *Gba^D409H/D409H^* (DH/DH) and *Gba^D409H/null^* (DH/null) animals. The staining was performed by Histoserv Inc. (Germantown, MD, USA) using their general histology protocols. Images were collected by Zeiss Axioscan (Thornwood, NY, USA) for (**a**) H&E staining in the liver (**top**) and spleen (**bottom**) over a broad region. Scale bars, 500 µm. (**b**) H&E staining in the liver (**top**) and spleen (**bottom**) at higher magnification. Scale bars, 50 µm.

To evaluate visceral pathology in the four *Gba1* mutant mice, liver and spleen were collected from 6-month-old mice and H&E staining was performed on the transverse section of each tissue. No Gaucher cells were observed in the liver or spleen in mice with any of the four *Gba1* genotypes. To determine whether additional maturation of the mice was needed for the development of pathological cells, we repeated the evaluations using 14-month-old mice. However, again, we did not detect Gaucher cells in the liver and spleen of the older mutant mice (Figure 2).

### 2.3. Evaluation of Motor Behavior in the L444P Gba1 Mutant Mice

**Figure 3:**
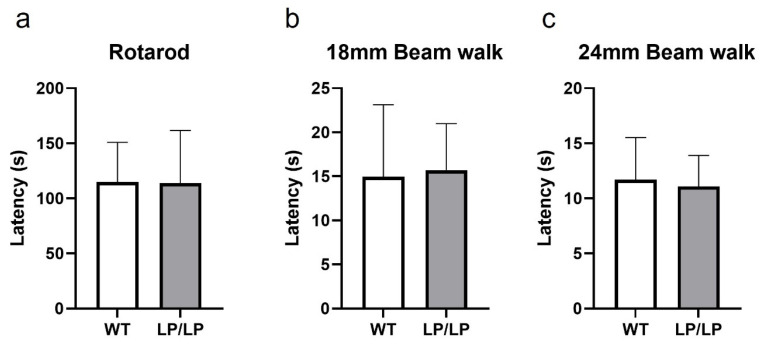
Evaluation of motor behavior in the *Gba^L444P/L444P^* mouse. (**a**) Rotarod testing. Latency until mice fail to walk on rotating rod was measured. (**b**) Balance beam testing using an 18 mm diameter beam. Latency to cross the entire beam was measured. (**c**) Balance beam test using a 24 mm diameter beam. Mice were tested at 16 months. WT is wildtype and LP/LP is *Gba^L444P/L444P^*. Ten mice of each genotype were evaluated. Error bars indicate the standard deviation. *t*-test was the statistical method used.

While motor symptoms can be seen in patients with nGD- and *GBA1*-associated PD, the assessment of the motor behavior of L444P and D409H homozygous mutant mice has not been previously reported. Since the L444P is a common *GBA1* mutation associated with PD, and the LP/LP mice showed both lipid accumulation and reduction in GCase protein levels (Figure 1), we tested the motor behavior of LP/LP mice compared with wildtype control mice. Motor balance was evaluated in 16-month-old mice using a rotarod assay, but no significant differences in the latency to stay at acclimated rotarod speed were detected between LP/LP and WT mice (Figure 3a). A balance beam test was also performed using both a narrow beam (18 mm diameter) and a wide beam (24 mm), but no differences were noted in time to cross the beam between two groups with either beam width (Figure 3b,c). The complete motor behavior testing data are available on Zenodo at https://zenodo.org/records/10430962 (accessed on 25 December 2023).

### 2.4. Evaluation of Neuropathology in the Gba1 Mutant Mice

**Figure 4:**
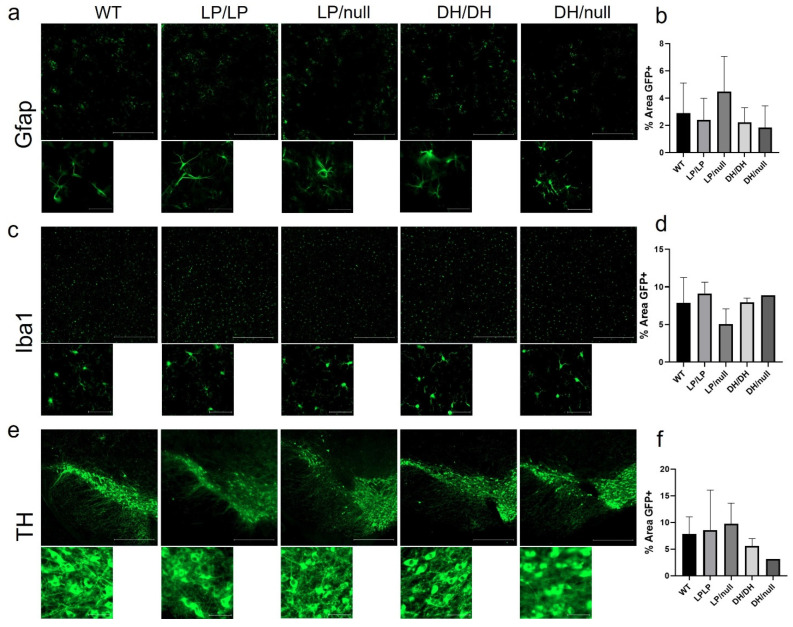
Evaluation of markers of neuropathology in brain from *Gba1* mutant mice. Immunohistochemistry was conducted on brain sections from14-month-old wildtype (WT), *Gba^L444P/L444P^* (LP/LP), *Gba^L444P/null^* (LP/null), *Gba^D409H/D409H^* (DH/DH) and *Gba^D409H/null^* (DH/null) animals. (**a**) Gfap staining for astrocytes in striatal sections of brain from mice with each genotype. (**b**) Quantification analysis of Gfap staining in mice with each genotype. For WT N = 4, LP/LP N = 3, LP/null N = 5, DH/DH N = 4 and DH/null, N = 2. (**c**) Iba1 staining for microglia in striatal sections of brain from mice with each genotype. (**d**) Quantification analysis of Iba1 staining in mice with each genotype. For WT N = 4, LP/LP N = 3, LP/null, N = 4, DH/DH, N = 2, DH/null, N = 1. No statistics are available for the D409H/null animals because data are only available for one animal. (**e**) Tyrosine hydroxylase (TH) staining for DA neurons in the substantia nigra of mice with each genotype. (**f**) Quantification analysis of TH staining in each genotype. For WT N = 3, LP/LP N = 2, LP/null N = 3, DH/DH N = 2 and DH/null, N = 1. Scale bars in the upper images represent 500 µm and in the lower images are 50 µm. Error bars indicate the standard deviation. One-way ANOVA with Sidak’s multiple-comparison test was the statistical method used.

Since astrogliosis and microgliosis are common markers of CNS pathology detected in nGD- and *GBA1*-associated PD, striatal tissue isolated from brain samples of aged mice with each genotype group were stained for Gfap and Iba1, respectively, and quantification of intensity was evaluated on the regions stained with each protein marker. While relatively higher levels of fibril staining of Gfap was detected in LP/null mice compared to the other genotypes, the differences did not reach statistical significance (Figure 4a,b). Also, there was no significant differences in Iba1 staining between five genotype groups in striatal sections (Figure 4c,d). We were also not able to find any differences in cellular morphology in the Gfap- and Iba1-stained cells regardless of genotype (Figure 4a,c). Midbrain sections harboring substantia nigra were also stained for tyrosine hydroxylase to evaluate dopaminergic neuronal loss, but no significant differences were detected between five genotypes (Figure 4e,f). All the immunohistochemistry quantification data are available on Zenodo at https://zenodo.org/records/10431016 (accessed on 25 December 2023).

**Figure 5:**
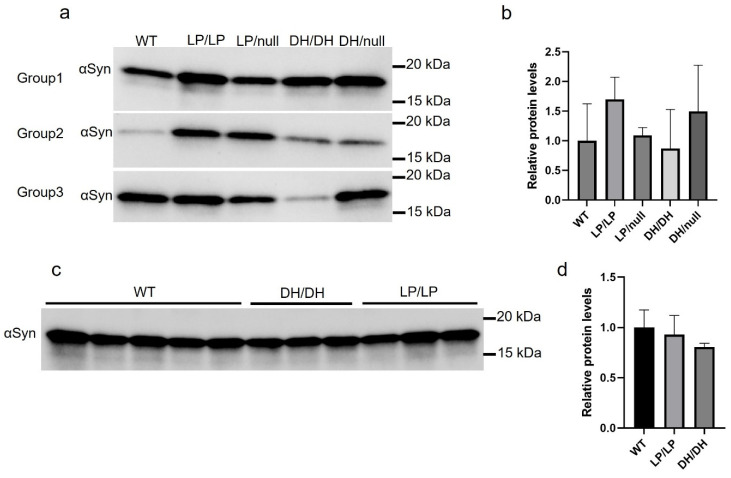
Evaluation of alpha-synuclein levels in brain samples from *Gba1* mutant mice. Immunoblotting was conducted in brain lysates from wildtype (WT), *Gba^L444P/L444P^* (LP/LP), *Gba^L444P/null^* (LP/null), *Gba^D409H/D409H^* (DH/DH) and *Gba^D409H/null^* (DH/null) animals. All protein lysates were extracted from coronally cut mouse brain pieces harboring the cerebral cortex, basal ganglia, hippocampus and midbrain. (**a**) Immunoblotting for total alpha-synuclein in RIPA-brain tissue lysates from 6-month-old animals. Size of band is approximately 15 kDa. The experiment was repeated in three different groups of animals including mice with each genotype. (**b**) Quantification of immunoblot data from 6-month-old animals. (**c**) Immunoblotting for total alpha-synuclein in RIPA-brain tissue lysates from 14-month-old animals. Experiment was repeated in 5 wildtype mice and 3 different DH/DH and LP/LP mice. (**d**) Quantification of immunoblot data from 14-month-old animals. Error bars indicate the standard deviation. One-way ANOVA with Sidak’s multiple-comparison test was used for statistics.

We evaluated the extent of α-synuclein levels in brain extracts of young adult (6-month-old) and older adult (14-month-old) mice. There was a trend toward greater accumulation in LP/LP and DH/null models compared to wildtype in the young adult mouse group, but this did not reach statistical significance (Figure 5a,b). There were no significant differences in α-synuclein levels between wildtype, DH/DH and LP/LP mice at 14 months (Figure 5c,d). All the immunoblot quantification data are available on Zenodo at https://zenodo.org/records/10431048 (accessed on 25 December 2023).

## 3. Discussion

The four different *Gba1* knock-in mice (LP/LP, LP/null, DH/DH and DH/null) evaluated as murine models of GD- or *GBA1*-associated PD did not exhibit overt phenotypic manifestations encountered in these disorders despite having reduced glucocerebrosidase activity and some accumulation of glucosylsphingosine. Our findings confirm previous studies indicating that murine models of Gaucher disease show very mild to no neurological manifestations even though they exhibit reduced glucocerebrosidase activity [13,19,22]. The *Gba1* mutant mice did not differ in behavior, longevity or fertility from age and sex-matched wildtype mice with the same genetic background. This observation suggests that the introduction of an analogous *Gba1* mutation is insufficient to induce the disease pathology observed in patients with GD.

The GCase activity in brain and liver from LP/LP and DH/DH mice was about 20–25% and 5–10% of wildtype, respectively, which, overall, is consistent with previous reports, although it was surprising to see that the DH/DH mice showed lower activity than LP/LP mice, as this is not what is generally observed in patients with these genotypes [14,19,23]. Increased levels of GlcSph were detected in both brain and livers of the LP/LP and DH/DH mice, while there was no change in GlcCer levels, consistent with previous reports [15,22,24]. The GCase activity was lower in liver than brain, corresponding to higher amounts GlcSph in liver than in brains in both L444P and D409H mice. As reported in previous studies, the LP/LP mice had reduced GCase protein levels [25]. However, the previously reported reduced GCase levels described in DH/DH mouse brains [26] were not found in our study, and hence, further replication studies are necessary to confirm the impact of the D409H mutation on GCase protein levels. Interestingly, D409H mutation carriers showed higher levels of GlcSph than L444P mutation carriers, while GCase activity was similar between the two. Mice with a combination of a null allele and missense mutation, as expected, had lower GCase activity levels than their homozygous counterparts, consistent with the finding that GlcSph levels in LP/null and DH/null were higher than that of LP/LP and DH/DH mice. However, despite the strong reduction in GCase activity and increased glycolipid accumulation, neither LP/null nor DH/null mice exhibited the predicted pathological findings.

Remarkably, we did not observe histopathological changes in visceral tissues in any of the four *Gba1* mutant lines, even with aging. Previous studies described rare Gaucher cells in the liver and spleen of both LP/LP and DH/DH mice [18,19]. One possible explanation could be differences in strain background and cage conditions between studies. The DH/DH mice in our study may have a higher C57BL/6 (BL6) background ratio because the imported strain was rederived in the BL6 mice and backcrossed with the BL6 mice, while the DH/DH strain used in the previous study had a mixed background [19]. It is also possible that other investigators similarly did not find the expected phenotype, but they did not report their negative findings. Additionally, none of these *Gba1* models displayed neuroinflammation or other neuropathological findings. A previous study showed increased levels of astrocytes and alpha-synuclein in the striata of LP/LP mice [18], which again was not replicated in our data. This could be due in part to our limited sample size, which limited the statistical quantification of the neuropathological images. It is also possible that the age at testing (14 months) is still not sufficient to enable detection of neuropathological phenotypes associated with neurodegeneration.

Another potential limitation is that the GCase activity assay used in this study was performed on whole tissue extract, and thus did not reflect what was specifically occurring in the lysosome. To better understand the impact of lysosomal GCase, newer GCase probes that function exclusively in the lysosome could potentially be used [27]. Another approach would be to breed our lines with lyso-tag mice engineered to carry a tag for lysosomal immunoprecipitation for tissue-specific isolation of intact lysosomes [28,29].

The conditional *Gba1* knock-out mice reported in Enquist’s two previous studies showed either infiltrations of Gaucher cells in visceral organs [30] or acute neuronopathic symptoms [9]. The major biochemical difference between the conditional *Gba1* knock-out models and the mice presented in the current study is that GlcCer accumulation is only observed in the *Gba1* knock-out mice. The DH/null mice had a substantial increase in GlcSph but not GlcCer in brain and liver, and they did not develop neurodegeneration nor storage cell infiltrations. These results suggest that the accumulation of GluCer may have a greater impact on pathology. Another possible explanation for the vast phenotypic differences between knock-out and knock-in mice is that *Gba1* could have another function related to cell homeostasis, perhaps through interaction with other proteins. The levels of *Gba1* expression present in the knock-in models could be adequate to avoid disrupting the second function, thus explaining the lack of pathological findings.

While it was reported that the *GBA1* mutations associated with neuronopathic GD, such as L444P, accelerate disease progression and severity in *GBA1*-PD [31], our current study does not show that mice carrying these mutations accurately recapitulate phenotypes detected in patients with GD-PD. However, even among patients with GD, the penetrance of PD is extremely low, and other factors likely contribute to the risk. In this study, since the *Gba1* mutations resulted in significantly increased GlcSph levels but did not result in pathological changes, even in aged mice, it appears that in the mouse, GluSph accumulation alone is not sufficient to generate PD phenotypes, including the loss of dopaminergic neurons and alpha-synuclein accumulation observed in patients with PD [32]. Another possibility is that the lysosomal accumulation of glycolipid in mice is below the threshold required for the alpha-synuclein aggregation cascade, which results in neurodegeneration [32]. Others have shown that in order to generate mouse models of *GBA1*-PD, it has been necessary to introduce a secondary burden such as under-expression of prosaposin [22], over-expression of pathological alpha-synuclein [26] or injection of pre-formed fibrils [24].

While the conditional knock-out mice are useful models for the development of therapeutics such as gene therapy [9,30], they cannot be used to validate other strategies like small-molecule chaperones [33]. Thus, further studies and new strategies are necessary to develop more appropriate animal models of GD with GD-causal missense mutations.

## 4. Materials and Methods

### 4.1. Mouse Lines

*Gba^L444P^* mice were generated as previously described [18] (RRID:IMSR_JAX:024574). The details of the procedures used to generate the *Gba^L444P^* strain are also described at https://www.protocols.io/view/generation-of-gba-l444p-mutant-mouse-5jyl8p8qdg2w/v1 (accessed on 19 December 2023). Subsequently, the neo cassette was removed using Cre driver. Sperm from D409H mice [22,34] (RRID:MGI:7567849) was kindly provided by Dr. Ying Sun’s lab at Cincinnati Children’s Hospital Medical Center, and live mice were rederived in the NHGRI Transgenic Mouse Core Facility by injecting sperm into C57BL/6 females (RRID:IMSR_JAX:000664). Homozygous mutants were generated by inbreeding of heterozygotes. Haploinsufficient mice (*Gba*^+/−^) [8], originating from the *Gba1* knock line (RRID:IMSR_JAX:003321), were bred with L444P and D409H homozygotes to produce L444P/null (RRID:MGI:7567793) and D409H/null (RRID:MGI:7567850) animals. The total number of mice used in the study was 74 (WT: 24, LP/LP: 21, LP/null: 10, DH/DH: 12, DH/null: 7). All housing and breeding of mice were performed under NHGRI Animal Care and Use Committee-approved protocols.

### 4.2. Motor Behavioral Evaluations

The rotarod test and beam-walk assay were used to evaluate the motor behavior of the different mice. Prior to rotarod testing, mice were acclimated to standing on a non-rotating rod (Rotamex 5, Columbus Instrument, Columbus, OH, USA) for three 1-min trials. Three hours later, mice were acclimated to walking on the rod over three 90-s trials at a speed of 4 rpm. After training, the instrument was set to begin at 4 rpm and gradually increase by 1 rpm every 8.33 s to a maximum speed of 40 rpm over 5 min. Mice were placed on the rod and the latency was recorded for three trials daily for two days. For the beam-walk assay, mice were trained to cross beams of 24 mm and 18 mm in diameter. During training (over 2 days), each mouse crossed both beams three times. During the experimental day, mice crossed both beams twice per trial, for three trials. Latency to cross the beam was tracked with a stopwatch, and the number of foot slips during each crossing was counted. The details of the rotarod test and beam-walk assay procedures are described at https://www.protocols.io/view/motor-behavioral-evaluation-of-mice-x54v9p5kqg3e/v1 (accessed on 18 December 2023).

### 4.3. Tissue Collection

At age 14 months, mice with each of the five genotypes (WT, LP/LP, DH/DH, LP/null, DH/null) were anesthetized with Avertin (1 mL/25 g body weight) and perfused transcardially according to standard NIH protocol (Whole-Body Perfusion Fixation in Mice, v6, effective 27 August 2019) with 1× PBS (Quality Biological, Gaithersburg, MD, USA) containing 1 IU Heparin/mL, followed by 4% PFA for fixation. Brains were collected and post-fixed in 4% PFA at 4 °C for 24 h before being transferred to 30% sucrose for cryoprotection at 4 °C for 24–48 h, until the brain sank to the bottom of the vessel. The floating sections of brain were kept at 4 °C for 8 weeks maximum for immunohistochemistry. Spleen and liver samples were collected using the same perfusion procedure for histological evaluations. After 24 h post-fixation, tissues were kept in 70% ethanol until paraffin embedding. For the GCase activity assays, lipid analysis and immunoblotting, brain (left and right hemispheres) and liver were collected after performing euthanasia in a CO_2_ chamber, snap-frozen and stored at −80 °C. The details of the tissue collection procedures are described at https://www.protocols.io/view/mouse-tissue-collection-n2bvj3ejwlk5/v1 (accessed on 19 December 2023).

### 4.4. Immunohistochemistry (IHC)

Brain tissue was sliced with a freezing sliding microtome (ThermoScientific HM450, Waltham, MA, USA) at 30 μm and stored in 1× PBS with 0.1% sodium azide until needed. Selected sections were mounted on a slide (FischerBrand SuperFrost Plus, Waltham, MA, USA) and were dried at room temperature for 30 min. A hydrophobic barrier was created around the tissue sections with a PAP pen (Newcomer Supply, Middleton, WI, USA). Slides were rehydrated in 1× PBS for at least 10 min before blocking for 1 h at room temperature in a solution of 15% Normal Goat Serum (Vector Laboratories, Newark, CA, USA) in 1× PBS with 2% Tween-20 (PBS-T). Diluted (10% Normal Goat Serum in PBS-T) primary antibodies (Gfap 1/1000, Proteintech 16825-1-AP, RRID:AB_2109646; Iba1 1/400, Abcam ab178846 (Cambridge, UK), RRID:AB_2636859; TH 1/400, Sigma-Aldrich AB152 (St. Louis, MO, USA), RRID:AB_390204) were incubated overnight in a humidity chamber at 4 °C and rinsed three times for 5 min with 1× PBS. Secondary antibody (1/400 Alexa-488, Abcam ab150077, RRID:AB_2630356) diluted in 10% Normal Goat Serum in PBS-T was added to the slides and incubated at room temperature for 2 h. Slides were rinsed three times in 1× PBS for 5 min then topped with a coverslip (FischerBrand) with DAPI antifade medium (Vector Laboratories) and left at room temperature until dry (usually overnight). The details of IHC procedures are described at https://www.protocols.io/view/immunohistochemistry-using-floating-section-6qpvr36yovmk/v1 (accessed on 1 August 2023).

### 4.5. IHC Data Processing

Widefield fluorescent images were collected for GFP and DAPI fluorescent channels using a Zeiss AxioScan.Z1 slide scanning microscope system (Carl Zeiss Inc., Thornwood, NY, USA) with a Plan-Apochromat 20×/0.8 objective lens. All images were acquired using a Orca flash 4.0 camera (Hamamatsu Photonics, Shizuoka, Japan), with an average tile count of 165 tiles per brain section. The Zeiss ZEN blue v.2.3 software package (https://www.zeiss.com/microscopy/en/products/software/zeiss-zen-lite.html#contact (accessed on 25 December 2023), RRID:SCR_013672) was used for collection and stitching of the 2-color (DAPI and GFP) tiled images. Widefield fluorescent images were then post-processed using MediaCybernetics’ Image-Pro v.11 software package (https://mediacy.com/image-pro (accessed on 25 December 2023), RRID:SCR_016497). Every stitched image was processed using a protocol modified for each antibody. Initially, the image was masked to solely include the tissue in areas of interest. Then, Smart Segmentation was used to separate GFP-expressing puncta or fibrils from DAPI-stained nuclei in tissue evaluated with Gfap and anti-TH antibodies. Threshold segmentation was used for Iba1 staining to separate actual signal from background autofluorescence. Finally, the Count/Size function was designed to extract the percent of the tissue sample that stained positive for GFP. The details of IHC data processing procedures are described at https://www.protocols.io/view/immunohistochemistry-data-processing-x54v9p5omg3e/v1 (accessed on 19 December 2023).

### 4.6. GCase Activity Assays

GCase buffer (20 mL of 0.2M Na_2_HPO_4_ with 15 mL of 0.1 M citrate adjusted to pH 5.4 with additional citrate as needed) was made and activated on the day of the assay (one Roche cOmplete mini protease inhibitor tablet (Sigma-Aldrich, St. Louis, MO, USA) per 10 mL buffer, 0.25% Triton X-100 and 0.2% sodium taurocholate). Protein lysates were prepared in a 96-well plate, diluting samples with GCase buffer. The concentration of protein was determined via a BCA assay and control and experimental samples were maintained at similar concentrations (0.5–1 mg/mL). A total of 0.8 mM CBE was prepared by diluting 100 mM CBE in GCase buffer. Control buffer without CBE was also prepared by mixing DMSO with GCase buffer (9.2:0.8 GCase buffer: DMSO). Then, 10 µL of protein lysate from the 96-well plate was pipetted to each assay well in a 384-well plate with four replications using an Eppendorf multichannel pipette. Total volumes of 5 µL of 0.8 mM CBE and 5 µL of control buffer without CBE were added and incubated for 15 min at 37 °C, with shaking at 600 rpm. A total of 2.5 mM 4-methylumbelliferyl-B-D-glucoside (4-MU) was then prepared by diluting 1M 4-MU in GCase buffer. Following the 15 min incubation, the 384-well plate was spun down and 15 µL of 4-MU solution was added to each assay well to reach a total volume of 30 µL per well and incubated for 1 h at 37 °C, shaking at 450 rpm. Following incubation, plates were spun down and 30 µL of 1M glycine solution (pH 10.5) was added to each assay well. 4-MU fluorescence was read with a microplate reader (Flexstation 3, Molecular Devices, San Jose, CA, USA; excitation: 365 nm; emission: 449 nm; cutoff: 435 nm; 3 reads/well). GCase activity was calculated for each protein lysate according to the equation: (fluorescence of CBE-free sample minus fluorescence of sample with CBE)/protein concentration. The details of the GCase activity assay are described at https://www.protocols.io/view/in-vitro-gcase-activity-assay-dm6gp3b7dvzp/v1 (1 August 2023).

### 4.7. GluCer and GluSph Assays

The mouse brain and liver tissues (100–300 mg) were homogenized in 2% CHAPS solution (4 mL/g wet tissue) in 2 mL Omni homogenization tubes containing 8 mm ceramic beads. The homogenates were processed on the Bead Ruptor 24 (Omni International, Kennesaw, GA, USA) for two 30 s cycles at 5.65 m/s with a 45 s pause time. The lipids glucosylsphingosine and glucosylceramide in the homogenate (50 μL) were extracted and analyzed using the liquid chromatography with tandem mass spectrometry method as previously described [35]. The details of the lipid analysis are described at https://www.protocols.io/view/glucosylceramide-and-glucosylsphingosine-analysis-261gedk8wv47/v2 (accessed on 20 December 2023).

### 4.8. Immunoblotting

Protein lysates in RIPA buffer were treated with protease inhibitor and phosphatase inhibitor and diluted using the sample RIPA buffer to ensure equal concentrations for loading. A total of 4× Leammli sample loading buffer (Biorad, Hercules, CA, USA) with 10% β-mercaptoethanol was mixed with protein lysates and boiled at 99 °C for 5 min. A total volume of 20 µL of each sample was loaded into TGX precast gels in Tris-Glycine-SDS buffer, along with 10 µL of protein standard, and run for one hour at 120 V. Proteins were transferred to PVDF membranes using a Trans-Blot Turbo transfer system (Biorad). The membrane was dried for 30 min, reactivated with 100% MeOH and rinsed with RO water. Ponceau S stain was added for 5 min and rinsed with RO water prior to imaging total proteins to normalize the data. After washing the membrane with buffer (1× TBS + 0.1% Tween 20) for 10 min, the membrane was incubated with blocking solution (2.5% milk mixed with 0.5× Intercept blocking buffer (LI-COR, Lincoln, NE, USA) in TBS) for 2 h while shaking. Primary antibody solution was prepared by diluting the antibody with the antibody dilution solution (2.5% milk mixed with 0.5× Intercept antibody diluent (LI-COR) in TBS). Blocking solution was replaced with antibody solution, and incubated overnight at 4 °C. The membrane was washed three times for 10 min with a wash buffer. Secondary antibody at the same antibody dilution buffer concentration was incubated with the membrane for 1 h on the shaker at RT. After washing three times for 10 min, membranes were stained with ECL solution (SuperSiganal West Pico PLUS, Thermo Scientific) for 5 min and imaged with the Chemidoc MP (Biorad). Quantification of the images was performed using Image Lab software v.6.1 (https://www.bio-rad.com/en-us/product/image-lab-software?ID=KRE6P5E8Z (accessed on 25 December 2023) RRID:SCR_014210). The details of the immunoblotting procedures are described at https://www.protocols.io/view/western-blotting-3byl4qbnrvo5/v1 (accessed on 1 August 2023)

### 4.9. Statistics

All experimental data were averaged for each genotype group and compared between groups using the statistical methods specified here. For the GCase activity assay, lipid assay, IHC and immunoblot results, a one-way ANOVA test with Sidak’s multiple-comparison adjustment was used to obtain a statistical *p*-value for pairwise comparison of each group. For the motor behavioral evaluations, an unpaired *t*-test was used to compare the average of the two genotype groups. GraphPad PRISM v.9.5 (https://www.graphpad.com/features (accessed on 25 December 2023, RRID: SCR_002798) was used for all the statistical analyses.

## Data Availability

The quantification data presented in this study are openly available on the Zenodo repository (https://zenodo.org (accessed on 25 December 2023)) with URLs cited in each related paragraph of the manuscript.
